# Preventive Effects of *Citrus unshiu* Peel Extracts on Bone and Lipid Metabolism in OVX Rats

**DOI:** 10.3390/molecules19010783

**Published:** 2014-01-09

**Authors:** Dong Wook Lim, Youngseok Lee, Yun Tai Kim

**Affiliations:** 1 Functionality Evaluation Research Group, Division of Metabolism and Functionality Research, Korea Food Research Institute, Seongnam 463-746, Korea; 2 Department of Bio & Fermentation Convergence Technology, Kookmin University, Seoul 136-702, Korea

**Keywords:** *Citrus unshiu* peel, anti osteoporosis, hepatoprotective effect

## Abstract

Dried *Citrus unshiu* peel has been widely used for various medicinal purposes in Oriental Medicine. This study evaluated the metabolic effects of dried *C. unshiu* peel in ovariectomized (OVX) rats. The OVX rats were divided into five groups treated with distilled water, 17β-estradiol (E2 10 μg/kg, once daily, i.p.) and dried *C. unshiu* peel extracts (DCPE 30, 100 and 300 mg/kg, once daily, p.o.) for eight weeks. The treatments with high-dose DCPE significantly decreased the bone mineral density (BMD) loss in the femur, which was reflected by the decrease in alkaline phosphatase (ALP), telopeptides of collagen type I (CTx) and osteocalcin (OC) serum levels. It also inhibited the increase in lipoprotein levels compared to the OVX-control group without elevating the serum levels of estradiol, aspartate aminotransferase (AST) and alanine transaminase (ALT). Furthermore, DCPE exhibits a hepatoprotective effect in OVX-induced hepatic steatosis, indicated by reduced hepatic lipid contents. Taken together, our findings suggest that DCPE has the potential to improve both lipid and bone metabolism without influencing hormones such as estrogen in OVX rats.

## 1. Introduction

Menopause is often associated with serious public problems in middle-aged women; thus, appropriate care and management of health after menopause is important for maintaining a woman’s quality of life [1]. A reduction in estrogen levels is commonly believed to cause psychological and mood changes, as well as physiological changes that result in symptoms such as osteoporosis, breast cancer, hot flashes, obesity, hyperlipidemia, and cardiovascular disease [2]. These menopausal symptoms can be at least comparatively reversed by the local or systemic administration of exogenous estrogens; thus, hormone replacement therapy (HRT) has been used to ameliorate menopausal symptoms [3]. However, long-term HRT increases the risk of several serious diseases, such as breast and endometrial cancer, thromboembolic events and vaginal bleeding [4]. Therefore, alternative approaches, including dietary interventions, have been interested in the treatment of menopausal symptoms.

Phytoestrogens are naturally occurring hormone-like compounds found in plants that can be subdivided into coumestans, isoflavonoids and lignans. Coumestrol, genistein, daidzein, and their plant precursors, are mainly found in soybeans and clover [5]. Isoflavones, especially derived from plants have various biological activities. They can improve metabolic symptoms [6] and bone-protective effects [7] by menopause. Although some studies have suggested that isoflavones have potential health benefits in postmenopausal women [8], a number of randomized trials have also failed to show a sustained enhancement of bone by isoflavone supplements. Since the evidence to support any beneficial effect of isoflavone intake on menopausal symptoms is not clear, making it is necessary to investigate the effects of natural agents other than isoflavones [9].

*Citrus unshiu* Marcov, which belongs to the family of Rutaceae, is a seedless and easy-peeling Korean citrus fruit that accounts for 30% of the total fruits produced in Korea. Its dried peels have been widely used as a folk medicine in Korea, China and Japan to improve bronchial and asthmatic conditions or blood circulation [10,11]. Dried *C. unshiu* peel has also been reported to process anti-inflammatory [12], anti-obesity [10], anti-allergic [13], hypolipidemic [14] and anti-cancer [15] effects. *C. unshiu* peel contains many phytochemicals such as hesperidin, naringin and nobiletin [16]. In particular hesperidin, the most abundant flavonoid in *Citrus* peel [17], has been shown to prevent bone loss in male orchidectomized rats following citrus juice consumption [18]. These reports led us to hypothesize that *C. unshiu* peel extracts could serve as a potent therapeutic agent for the menopausal symptoms, which often include dyslipidemia and osteoporosis. However, their efficacy needs to be scientifically evaluated *in vivo* experiments.

Here, we used OVX rats, which exhibit most of the characteristics of human menopausal symptoms [19]. They were treated with standardized dried *C. unshiu* peel extracts (DCPE) during an eight-week experimental period and then assessed the metabolic parameters related to menopausal symptoms. 

## 2. Results and Discussion

### 2.1. Analysis of Hesperidin from Dried Citrus unshiu Peel Extracts

**Figure 1:**
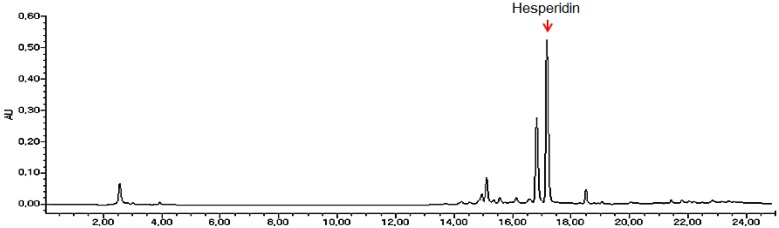
2-D HPLC chromatogram for standardization of DCPE.

DCPE was monitored at 284 nm for hesperidin (Figure 1). The content of hesperidin was calculated for standardization. *C. unshiu* peel extracts was standardized to contain 21.7 mg/g hesperidin.

### 2.2. Effect of DCPE on Body Weight and Serum Lipid Concentrations in OVX Rats

**Figure 2:**
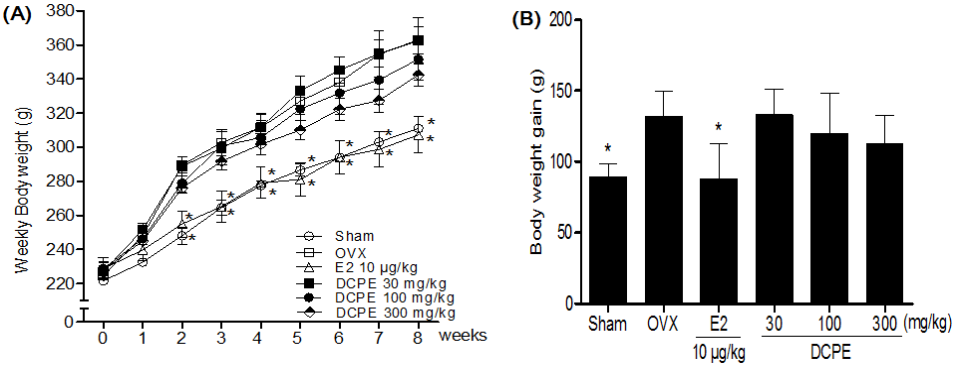
Effects of DCPE on weekly body weight (**A**) and body weight gain after eight weeks (**B**) in OVX rats. All data are the mean ± SD (*n* = 12 per group). The asterisks indicated significant differences from the OVX-control group (* *p* < 0.05) based on one-way ANOVA and the Turkey post hoc test.

The body weights in the OVX-control group statically increased compared to the sham group. As expected, a significant difference in body weight was observed between the E2 10 μg/kg treated group and the OVX-control group by two weeks after initiating administration (Figure 2A). However, there was no significant difference in the body weight gain of the DCPE treated groups during the experimental period (Figure 2B). After eight weeks of treatments, the DCPE 300 mg/kg treated group showed significantly lower serum TC, TG and LDL-c levels, while causing the reverse on serum HDL-c. Similarly, DCPE 300 mg/kg caused significant reductions in the coronary artery risk and atherogenic indices (Table 1).

### 2.3. Effect of DCPE on OVX-Induced Fatty Liver

**Figure 3:**
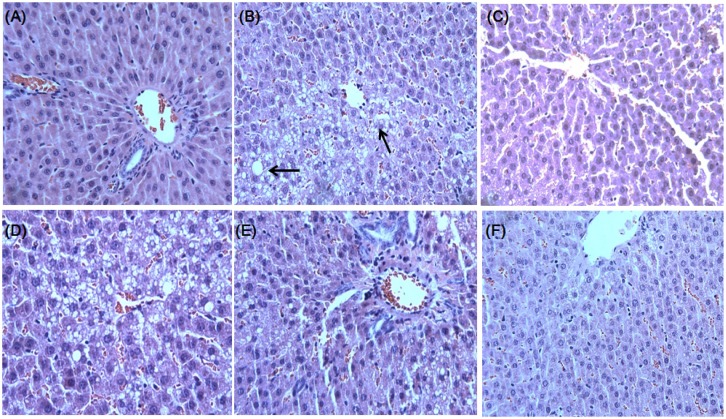
Histological images of liver tissues from OVX-induced hepatic steatosis displaying the hepatoprotective effects of DCPE. There were no histological abnormalities observed in sham rats (**A**). OVX-induced hepatic steatosis showing hepatocytes with severe cytoplasmic vacuoles and swelling (**B**). E2 10 μg/kg resulted in the prevention of hepatic fatty deposition in hepatocytes (**C**). The treatments with DCPE 30, 100 and 300 mg/kg resulted in the prevention of hepatic fatty deposition in hepatocytes (**D**, **E** and **F**). The tissues were surgically excised and subjected to histological study by staining with hematoxylin and eosin (H & E). The arrows indicate fatty hepatocytes.

We found that DCPE exhibits a hepatoprotective effect *in vivo*, indicated by reduced hepatic lipid contents, serum AST and ALT levels (Table 2). It was found that OVX-control rats developed a high degree of steatosis, with severe cytoplasmic vacuoles and hepatocyte swelling (Figure 3B), whereas no histological abnormalities were observed in normal rats (Figure 3A). The treatments of DCPE 300 mg/kg resulted in the prevention of hepatic fatty deposition in hepatocytes (Figure 3F).

**Table 1:** Effects of DCPE on serum lipoproteins in OVX rats.

Groups	TC	TG	HDL-c	LDL-c	CRI	AI
	(mg/dL)	(mg/dL)	(mg/dL)	(mg/dL)		
I	75.8 ± 8.5	78.5 ± 5.2	50.5 ± 4.1	15.7 ± 6.1	1.7 ± 0.2	0.3 ± 0.2
II	97.4 ± 5.1 ^c^	98.3 ± 6.4 ^c^	41.5 ± 2.3	33.6 ± 4.2 ^c^	2.3 ± 0.3 ^c^	0.8 ± 0.2 ^c^
III	90.0 ± 4.8	85.7 ± 8.7^ a^	44.8 ± 4.3	18.3 ± 5.4^ a^	2.0 ± 0.2^ a^	0.4 ± 0.1^ a^
IV	98.2 ± 5.8	90.2 ± 5.4	42.5 ± 3.8	27.3 ± 4.2	2.3 ± 0.3	0.6 ± 0.1
V	92.0 ± 5.4	88.7 ± 8.7	42.5 ± 4.8	26.3 ± 5.4	2.2 ± 0.2	0.6 ± 0.2
VI	85.0 ± 6.5 ^a^	80.9 ± 5.6 ^a^	48.9 ± 4.6	17.9 ± 4.8 ^a^	1.7 ± 0.2 ^a^	0.4 ± 0.3 ^b^

Group I = Sham; Group II = OVX-control; Group III = E2 10 µg/kg; Group IV = DCPE 30 mg/kg; Group V = DCPE 100 mg/kg; Group VI = DCPE 300 mg/kg. Data are mean ± SD (*n* = 12 per group). ^a^ A significant decrease at *p* < 0.05, when compared with Group II values; ^b^ A significant decrease at *p* < 0.01, when compared with Group II values; ^c^ A significant increase at *p* < 0.05, when compared with Group I values. CRI: coronary artery risk index, AI: atherogenic index.

**Table 2:** Effects of DCPE on OVX-induced hepatic steatosis.

Groups	Serum (mg/dL)		Liver lipid (mg/g wet wt)	
	AST	ALT	TC	TG
I	115.1 ± 11.6	62.1 ± 6.9	6.1 ± 0.7	38.2 ± 5.9
II	144.4 ± 25.7 ^c^	96.5 ± 6.6 ^c^	10.1 ± 0.7 ^c^	69.8 ± 6.4 ^c^
III	121.2 ± 17.4^ a^	72.5 ± 5.6^ a^	7.0 ± 0.5 ^a^	54.5 ± 6.2 ^a^
IV	132.3 ± 16.4	84.5 ± 6.6	8.2 ± 0.6	70.5 ± 4.2
V	123.1 ± 16.4 ^a^	75.3 ± 4.9^ a^	7.2 ± 0.6 ^a^	52.8 ± 6.8 ^a^
VI	118.6 ± 10.5 ^a^	67.5 ± 6.8 ^a^	6.5 ± 0.5 ^a^	44.1 ± 5.8 ^a^

The mean ± SD of all data (*n* = 12 per group) are shown. ^a^ A significant decrease at *p* < 0.05, when compared with Group II values; ^c^ A significant increase at *p* < 0.05, when compared with Group I values.

### 2.4. Effect of DCPE on BMD and Organ Weights in OVX Rats

**Figure 4:**
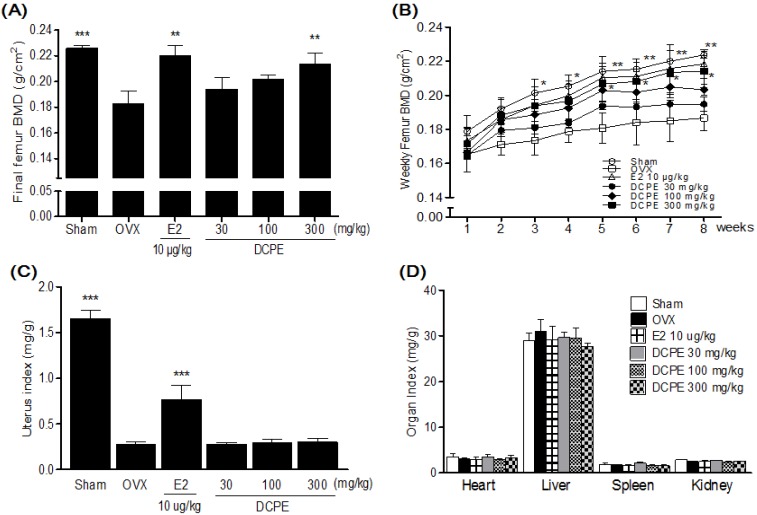
Effects of DCPE on final femur bone mineral density (BMD) (**A**), femur BMD changes (**B**), uterus index (**C**) and organ index (**D**). Data are the mean ± SD (*n* = 12 per group). * *p* < 0.05, ** *p* < 0.01 and *** *p* < 0.001, significant differences from the OVX-control group.

Three weeks after the OVX operation, OVX groups showed a significant decrease in the femur BMD compared to the sham group (Figure 4B). After eight weeks of treatments, the final femur BMD of the DCPE 300 mg/kg treated group was significantly higher than that of the OVX-control group (Figure 4A). OVX caused atrophy of uterine tissue, indicating the success of the surgical procedure and in the E2 10 μg/kg treated group, the uterus index (mg/g) increased significantly compared to the OVX-control group. However, the DCPE treated groups did not show an effect on the uterus index following OVX operation (Figure 4C). In addition, the index of heart, liver, spleen and kidney was not significantly different in each group; either (Figure 4D).

### 2.5. Effect of DCPE on Bone Marker in OVX Rats

**Figure 5:**
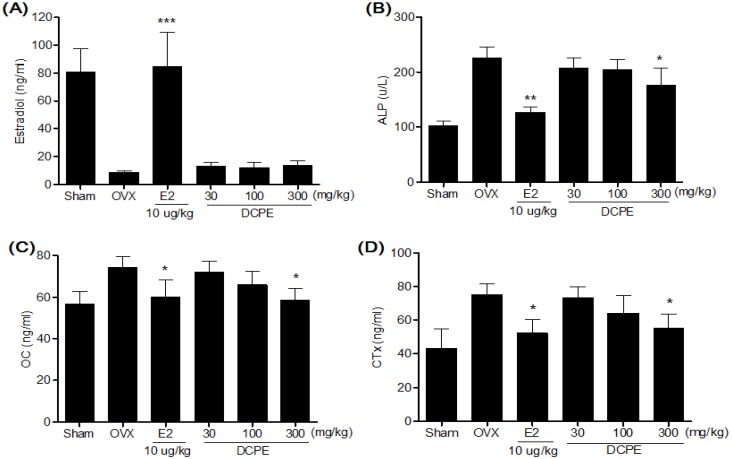
Effects of DCPE on serum estradiol (**A**), ALP (**B**), OC (**C**) and CTx (**B**) levels. Data are the mean ± SD values (*n* = 12 per group). * *p* < 0.05 ** *p* < 0.01 and *** *p* < 0.001, significant differences from the OVX-control group.

In our previous results [20], alkaline phosphatase (ALP), telopeptides of collagen type I (CTx) and osteocalcin (OC) levels in the OVX-control group were significantly higher compared to the sham group. After eight weeks of treatments, the DCPE 300 mg/kg treated group displayed significantly lower ALP, CTx and OC levels compared to the OVX-control group (Figure 5). In case of serum estradiol levels, the DCPE treated groups were not significantly different from the OVX-control group (Figure 5A).

### 2.6. Discussion

Our results demonstrate that the treatment with high-dose DCPE for eight-weeks significantly decreased the BMD loss in the femur and inhibited the increase in lipoprotein levels compared to the OVX-control group. These findings indicate that DCPE induced multiple metabolic changes in a rat model of postmenopausal symptoms.

Bone loss caused by estrogen deficiency in both experimental animals and humans is primarily due to an increase in osteoclastic bone resorption [21]. OVX rats, which display most of the characteristics of human postmenopausal osteoporosis [19] have been widely used as a model for the evaluation of potential osteoporosis treatments [22].

Like previous reports, our OVX resulted in significant decrease in the femur BMD after eight weeks. This BMD loss was accompanied by a significant increase in bone remodeling, as evidenced by the enhanced bone turnover makers. Elevated alkaline phosphatase (ALP) in serum levels, the most widely used biochemical bone turnover marker [23], was observed in OVX rats. Moreover, an increase of telopeptides of collagen type I (CTx), correlates with bone-resorption and bone formation marker osteocalcin (OC) in serum levels were observed in OVX rats. These results were supported by Hertrampf *et al.* [24]. The treatment with DCPE 300 mg/kg for eight week significantly decreased the BMD loss in the femur. It was reflected by the decrease in ALP, CTx and OC serum levels compared to the OVX-control group; this effect may have been caused by the decreased bone resorption.

Park *et al.* [11] found that supplementation with *C. unshiu* peel extract resulted in a significant decrease in body weight gain, body fat mass and blood glucose level in type 2 diabetic db/db mice. This suggestion was supported by the anti-obesity effects of *Citrus* peel extracts in a high fat diet-induced obesity *in vivo* model accompanied by regulation of the mRNA expressions of genes involved in adipogenesis in the adipose tissue [10,14]. However, in our results, there was no significant difference in the body weight gain of the DCPE treated groups in OVX rats. OVX dramatically increases body weights, while E2 treatment presents completely normal levels [25]. Although the mechanisms by which OVX induces an increase in body weight are not clear, estrogen deficiency induced body fat accumulation and subsequently caused an increase in body weight [26]. Estrogen may be directly involved in rat energy metabolism by binding to ER within the abdominal and subcutaneous fat tissues [27]. Estrogen affects its activities by binding to different estrogen receptors (ERs), ERα and ERβ. ERβ is more abundant than ERα in bone tissue while ERα is mainly distributed in reproductive cells. ERα is the dominant receptor mediating the most obvious effects of E2 in breast and uterus [28]. Oral administration of DCPE did not affect body weight gain, uterotrophic activity, and serum estradiol concentration in OVX rats. Consistent with our finding from the E2 treated group, the DCPE might have anti-osteoporotic effects in OVX rats, without the influence of hormones such as estrogen.

We found in current study that there was an obvious lipid metabolic disturbance in OVX rats. The TC and TG concentration in liver in OVX control group at eight weeks after surgery was found to be obviously higher than those in other groups. Although OVX increases TC and TG concentrations in liver, the increased insulin concentration in OVX rats may accelerate the dephosphorylation effect of the HMG-CoA reductase, which is a rate-limiting enzyme of the composition of cholesterol in liver [29]. Serum AST and ALT levels are clinically and toxicologically important indicators [30], and increase as a results of tissue damage caused by toxicants or disease conditions. It has also been reported that a high consumption of citrus flavonoids decreases the risk of coronary heart disease, given that naringin or hesperidin lowers serum cholesterol and triglycerides in rats [31]. Bok *et al.* [32] demonstrated that a mixture of naringin and hesperidin significantly lowered the levels of plasma and hepatic cholesterol and triglycerides as well as the HMG-CoA reductase activity in rats. In our results, DCPE caused no apparent liver toxicity, based on lower plasma AST and ALT. Moreover, DCPE exhibits a hepatoprotective effect, indicated with improved hepatic lipid contents in OVX rats. These results suggest that DCPE could regulate the lipid metabolic disturbance in liver in the OVX rats.

## 3. Experimental

### 3.1. Sample Preparation and HPLC Analysis

Dried peel of *C. unshiu* was purchased from the Kapdang Co. (Seoul, Korea). The sample was identified by Dr. HeeSoon Shin and a voucher specimen (#NP-1047) was deposited in the Functionality Evaluation Research Group, Korea Food Research Institute, Seoul, Korea. The dried peel of *C. unshiu* (300 g) was extracted separately with 70% ethanol (3,000 mL) for 3 h at 80 °C in a reflux apparatus. The extracts were filtered and concentrated under reduced pressure, and samples were lyophilized to yield a dark yellow powder. The yield of extracts was 20.1%. The standardization of *C. unshiu* peel extracts was performed by a high performance liquid chromatography (HPLC) system equipped with a Waters 1525 pump, a 2707 auto sampler and a 2998 photodiode array detector (PDA) detector. The separation was achieved at 30 °C on Waters Sunfire™ C18 (250 mm × 4 mm i.d., 5 μm particle size) column. *C. unshui* peel extracts was monitored at 284 nm for hesperidin. The run time was set at 25 min and the flow rate was 1.0 mL/min (sample injection volume was 10 μL). The mobile phases A and B were 1% H3PO4 (v/v) and CH3CN, respectively. The gradient elution was as follows: 0–10 min 0%–40% solvent B, 10–15 min 40%–100% solvent B, 15–25 min 100%–0% solvent B. *C. unshiu* peel extracts was standardized to contain 21.7 mg/g hesperidin.

### 3.2. Animals and Treatments

Female Sprague-Dawley (SD) rats, 8-weeks-old, were purchased from Samtako (Gyeonggi-do, Korea). Animals were housed two rats per cage in an air-conditioned room at 23 ± 1 °C, 55%–60% relative humidity and a 12 h light/dark cycle (07:00 lights on, 19:00 lights off) and were given a laboratory regular rodent diet. After acclimatization for 1 week, 9-week-old female SD rats were anesthetized with 2% of isoflurane and the ovaries were removed bilaterally. A sham operation, during which the ovaries were just touched with forceps, was performed on the sham group. One week after surgery, rats were divided into the six following treatment groups: sham + vehicle, OVX + vehicle, OVX + 17β-estradiol (E2, 10 μg/kg once daily, i.p.), OVX + DCPE (30, 100 and 300 mg/kg, once daily, p.o.). E2 was dissolved in distilled water, with 1% dimethyl sulfoxide (DMSO) and 0.1% Tween 20. All groups were treated for eight weeks. During the experimental period, body weight and femur bone mineral density (BMD) were determined weekly. At the end of the treatment period, the rats fasted for 12 h and blood was collected via the abdominal aorta under 2% of isoflurane anaesthesia. Uterus tissue and other organs were dissected, washed with saline solution and weighed for analysis. Uterus and organ indexes (mg/g) were calculated by dividing the uterus and organ weights by the body weight. Liver tissues were collected for the experiments described later in this section. All animal experiments were carried out according to the guidelines of the Korea Food Research Institutional Animal Care and Use Committee.

### 3.3. BMD Measurements

The BMD of femur was measured by a PIXImus (GE Lunar PIXImus, GE Healthcare, Madison, WI, USA), dual energy X-ray absorptiometer (DXA), equipped with appropriate software for bone density assessment in small laboratory animals. Calibration of the instrument was conducted as recommended by the manufacturer. Quality control with BMD (0.0553 g/cm^2^) and percentage fat composition (16.7%) of the phantom were also performed each time the instrument was switched on. All rats were placed in the same direction.

### 3.4. Serum Lipid, Estradiol and Bone Marker Analysis

The serum samples were prepared by centrifugation of the collected blood samples (1,013 g for 15 min at 4 °C), then stored at −80 °C for biochemical determinations. The serum concentrations of total cholesterol (TC), triglyceride (TG), high density lipoprotein-cholesterol (HDL-c), alanine transaminase (ALT), aspartate aminotransferase (AST) and alkaline phosphatase (ALP) were determined using an automatic analyzer (ADVIA 1650, Bayer, Tokyo, Japan). Serum LDL-c was estimated using Frieldwann’s equation. Serum hormone level was determined by radioimmunoassay (RIA). The estradiol RIA was performed according to the instructions accompanying a Coat-a-Count kit (Diagnostic Products, Los Angeles, CA, USA). Serum concentrations of the bone formation marker, osteocalcin (OC), were assayed using a rat ELISA kit (Metra OC, Quidel Corporation, San Diego, CA, USA). Serum levels of telopeptides of collagen type I (CTx), which correlate with bone resorption, with high levels indicating excessive osteoclastic activity, were analyzed using commercial ELISA kits (Serum CrossLaps, Nordic Bioscience, Herlev, Denmark; Metra Serum Pyd, Quidel Corporation). The hepatic lipid was extracted using the procedure developed by Folch *et al.* [33]. TC and TG contents were determined using a commercial enzymatic kit (Asan Pharm. Co., Seoul, Korea).

### 3.5. Liver Histological Analysis

The liver tissues were divided into the tissue freezing medium (TBS, Durham, NC, USA) and then deep-frozen in liquid nitrogen for their preservation. The liver tissues were cut into 5 μm-thick sections using a Cryostat instrument (CM 1850; Leica, Heidelberg, Germany). The tissue sections were fixed in 10% neutral phosphate-buffered formalin solution. The lipids and nuclei of the liver cells were stained with hematoxylin and eosin (H & E). A diagnosis of fatty liver was made based on the presence of macro- or micro-vesicular fat in >5% of the hepatocytes in a given slide.

### 3.6. Statistical Analysis

All data were presented as the mean ± standard deviation (SD). The effects of different treatments were compared by one-way ANOVA test, followed by the post-hoc Tukey_test for multiple comparisons using GraphPad Prism 5 (GraphPad Software Inc., La Jolla, CA, USA). *p* < 0.05 was considered statistically significant.

## 4. Conclusions

In conclusion, our results demonstrated that DCPE has multiple metabolic benefits in OVX rats. The treatment of DCPE led to decreased serum lipoprotein levels and significantly improved femur BMD, without elevating the serum levels of the liver enzymes, AST and ALT. Taken together, our findings suggest that DCPE has the potential to improve both lipid and bone metabolism without influencing hormones such as estrogen in OVX rats.
